# A Human-in-the-Loop Assistive Navigation Platform for UAS-Based Infrastructure Visual Inspection: System Architecture and Proof-of-Concept Demonstration

**DOI:** 10.3390/s26113615

**Published:** 2026-06-05

**Authors:** Martin Xu, Yuxiang Zhao, Zixin Wang, Mohamad Alipour

**Affiliations:** 1 Department of Electrical and Computer Engineering, University of Illinois Urbana-Champaign, Urbana, IL 61801, USA; martinx3@illinois.edu; 2 Department of Civil and Environmental Engineering, University of Illinois Urbana-Champaign, Urbana, IL 61801, USA; zhao132@illinois.edu (Y.Z.); alipour@illinois.edu (M.A.)

**Keywords:** infrastructure inspection, Unmanned Aerial Systems (UAS), assistive navigation, Augmented Reality (AR), 3D reconstruction

## Abstract

While Unmanned Aerial Systems (UAS) are increasingly used for infrastructure inspection, a critical gap exists between optimized path planning and reliable real-world execution. Fully autonomous flights face regulatory constraints and environmental risks, whereas manual piloting introduces inconsistencies that compromise data quality. To address this gap, this study proposes a human-in-the-loop assistive navigation platform that enables pilots to follow preplanned inspection trajectories while maintaining manual control. The proposed system integrates an Augmented Reality (AR)-based guidance module that provides real-time viewpoint localization with a mesh-coupled quality monitoring module that continuously evaluates view redundancy and triangulation uncertainty. A proof-of-concept field demonstration through an on-site façade inspection example indicates that the proposed platform has the potential to improve the consistency of viewpoint distribution, achieving closer adherence to planned spacing and stand-off distance. This results in more uniform spatial sampling, enhanced view redundancy, and reduced variability in theoretical uncertainty, leading to improved geometric conditions for Structure-from-Motion (SfM) reconstruction. Overall, the field demonstration highlights the potential of combining computational guidance with human decision-making to support reliable and high-quality UAS-based infrastructure inspection.

## 1. Introduction

The extensive and aging infrastructure systems in the United States pose increasing challenges to maintaining public safety and service reliability. Periodic visual inspection is essential for identifying structural deficiencies and preventing failures, while traditional manual approaches are often labor-intensive, costly, and constrained by complex or hazardous environments. Recent advances in robotics have accelerated the development of automated inspection solutions, among which UAS have emerged as a versatile and cost-effective platform. With their high mobility and sensing capabilities, UAS enable efficient data acquisition over large-scale assets and provide safe access to areas that are difficult or dangerous for human inspectors.

Due to these advantages, end-to-end UAS-based infrastructure visual inspection frameworks have been extensively studied in recent years for different tasks, including bridge inspection [1,2], building inspection [3,4], power system inspection [5], construction site inspection [6,7], and other types of civil infrastructure condition assessments [8,9]. These frameworks typically comprise three stages: mission planning, mission execution, and data analysis and reporting.

Among these stages, both mission planning and data analysis have been extensively studied. Many studies attempted to address mission planning for a wide range of environments, including buildings [10,11,12,13,14], bridges [15,16,17,18], and urban scenes [19,20,21,22]. Similarly, data analysis and reporting from UAS imagery have been actively explored, with recent advances in deep learning demonstrating strong potential for automated defect detection and assessment [23,24,25,26]. In contrast, mission execution remains relatively underexplored and is often over-simplified by assuming perfect localization and navigation.

Many studies assume fully autonomous execution enabled by precise Global Navigation Satellite System (GNSS) localization [27,28]. However, GNSS signals can be unreliable in environments such as urban canyons or beneath bridges [29]. Moreover, even with accurate positioning, unexpected obstacles or dynamic objects may arise that are not accounted for during mission planning. These challenges will introduce safety risks, particularly in inspection scenarios involving nearby traffic or pedestrians. Therefore, current regulatory frameworks, such as the Federal Aviation Administration (FAA) Part 107 [30], do not permit fully autonomous UAS operations without direct human supervision. A certified Remote Pilot in Command (RPIC) is required to maintain continuous operational control of the aircraft and always ensure safe operation [18]. Collectively, these technical and regulatory constraints make fully autonomous inspection currently impractical for many real-world deployments.

Alternatively, some approaches rely on pilots to manually plan and execute inspection missions [31,32], which makes it difficult to achieve high accuracy and often leads to deviations that compromise coverage and data quality, especially for infrastructure with complex geometries, where achieving complete and high-quality inspection is particularly challenging for human operators. These limitations highlight a critical gap between optimized mission planning and its reliable real-world execution, motivating the need for approaches that can effectively bridge this gap.

Augmented Reality (AR), which integrates virtual information with the physical environment in a spatially consistent manner, has emerged as a promising technology for enhancing human perception and interaction in complex tasks. By providing intuitive visual guidance and real-time situational awareness, AR has demonstrated the potential to improve task efficiency and accuracy. Motivated by these advantages, numerous studies have explored AR to assist UAS pilots in navigation, safety, and complex task execution. For example, AR interfaces have been developed to enable intuitive navigation and control through clicking, pointing, or voice commands [33,34,35], while other studies enhanced operational safety by improving situational awareness and hazard detection [36,37]. AR has also been applied to support complex tasks such as search operations, mission planning, and navigation in challenging environments, demonstrating improved efficiency and reduced cognitive load [38,39,40,41]. Although these studies focus on different applications, they collectively indicate that intuitive AR visualization can enhance task performance, highlighting the strong potential of AR to support pilots in cognitively demanding and operationally challenging UAS inspection tasks.

In recent years, AR has been increasingly adopted in various engineering and robotics applications, including engineering education [42], infrastructure maintenance [43], construction safety enhancement [44], and inspection training [45]. In the field of infrastructure inspection, the application of AR has also been explored and has demonstrated advantages from multiple perspectives. For example, Xu et al. [46] proposed an adaptive AR-based drone control interface to enhance safety during inspection while reducing pilot cognitive load. Diniz et al. [47] integrated power line detection with an AR interface to assist pilots in maintaining a safe distance during inspection, addressing the difficulty of visually perceiving thin power lines. These applications focus on applying AR interface to enhance safety, instead of the inspection quality.

In addition to safety, AR has been leveraged to enhance the inspection quality. Martins et al. [48] and Mascareñas et al. [49] developed AR-based platforms with defect labeling capabilities, projecting annotations onto Building Information Modeling (BIM) models to enable element-level recording and visualization. Lapointe et al. [50] used AR to improve the visualization of defects detected by deep learning models for bridge inspection, while Tan et al. [51] applied similar approaches for building exterior wall inspection. Although these studies demonstrated improvements in inspection accuracy and efficiency, they primarily focus on defect localization, visualization, and annotation after or during data collection. In contrast, limited attention has been given to guiding pilots during mission execution to ensure comprehensive inspection coverage and high-quality image acquisition.

To address this gap, a limited number of studies attempted to provide real-time feedback on data collection quality during mission execution to enhance the image capture quality. Wang et al. [52] integrated a rapid in-flight quality assessment function into the pilot interface, but it mainly evaluates image quality metrics such as blurriness and exposure, rather than overall 3D reconstruction quality, which depends on sufficient and stable viewpoint overlap at appropriate distances. Liu et al. [53] developed an AR interface to visualize reconstructed 3D models for immersive navigation and control. However, their approach is limited to visualizing the reconstructed point cloud to allow the pilot to subjectively adjust the inspection image collection process, instead of guiding the pilot to capture images that ensure inspection quality. Therefore, despite recent advances in AR-assisted inspection, there remains a lack of AR-based frameworks that provide real-time guidance to optimize image acquisition and improve reconstruction quality during UAS inspection missions.

To address these gaps and aid the execution of preplanned structural inspection tasks, we propose an assistive navigation and inspection platform to enable reliable human-in-the-loop infrastructure inspection during preplanned, optimized missions. The proposed platform integrates two key components: an AR-based navigation guidance module and an inspection progress monitoring and quality assurance module. The AR guidance module provides intuitive, real-time visual cues to assist pilots in accurately localizing target viewpoints and aligning camera orientations, thereby improving trajectory adherence and data acquisition quality. Complementarily, the monitoring and quality assurance module continuously evaluates inspection progress and data completeness based on the image capture history, enabling timely identification of coverage gaps and supporting informed decision-making during flight. A comparative experiment conducted on a building façade inspection task, comparing the platform against conventional manual inspection methods, provides evidence indicating that the system can help achieve closer adherence to the planned mission, enhances data consistency, and improves overall inspection quality and reliability.

## 2. Methodology

For this human-in-the-loop assistive navigation application, a modular system architecture was developed that integrates real-time telemetry, 3D coordinate transformations, and dynamic mesh visualization. The following sections detail the core components of the proposed methodology: the system architecture, coordinate transformations for viewpoint projection, the 3D reference model view redundancy and triangulation uncertainty estimation, and the human–computer interaction workflow.

### 2.1. System Architecture

**Figure 1:**
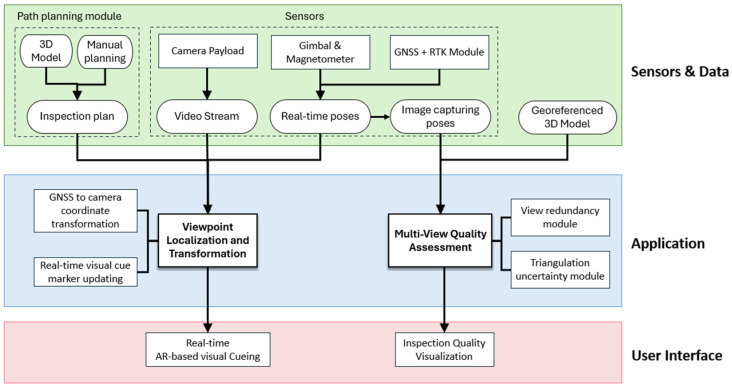
Block diagram of system architecture.

As illustrated in Figure 1, the system architecture comprises three modular layers: the sensors and data layer, the application layer, and the user interface (UI) layer. The sensors and data layer consist of both precomputed trajectory data generated by an independent path planning module and real-time video and positioning data acquired from onboard sensors. Prior to system operation, a separate path planning module is used to process the 3D model to generate a set of target viewpoints forming the inspection trajectory. This function can be achieved using various commercial or open-source software tools, such as Metashape [54], UgCS [55], and Mission Planner [56], or by devising a custom flight grid. During operation, the UAS provides a continuous stream of data: the camera payload delivers the live video feed, the gimbal and magnetometer provide camera attitude information (pitch, roll, yaw), and the GNSS/RTK module supplies global positioning data (latitude, longitude, altitude). In addition, a georeferenced 3D model serves as input, which can be obtained from existing BIM models, prior SfM reconstructions, or computer-aided modeling based on 2D drawings. These data streams, including the real-time video feed, pose estimates, precomputed trajectory viewpoints, and the 3D model, serve as inputs to the application layer.

The application layer is the computational core of this platform, which ingests the data from the sensors and data layer and processes them through two primary pipelines.

The Viewpoint Localization and Transformation pipeline, as introduced in Section 2.2, aims to calculate the position of the target viewpoint in the camera frame. Real-time position and attitude data are subscribed to the application layer, based on which the target viewpoint is transformed to the camera coordinate system and visualized on screen. In the Multiview Quality Assessment pipeline, as introduced in Section 2.3, the pose information of the captured images is recorded and employed to estimate the quality metrics, including view redundancy and triangulation uncertainty.

**Figure 2:**
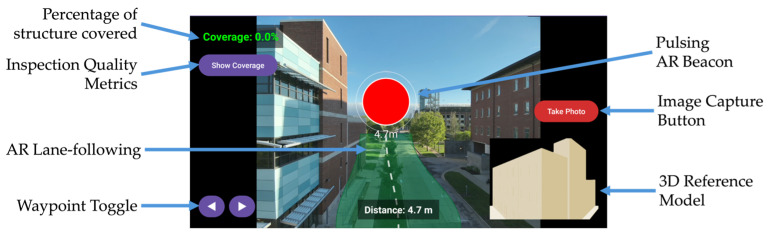
Complete UI with viewpoint projection in the middle, a synthetic 3D reference model in the bottom right, and additional buttons for pilot workflow.

The UI layer renders the processed data directly onto the screen of the remote controller, as seen in Figure 2. The Real-time AR-assisted Visual Cueing module generates multiple forms of intuitive guidance, including dynamic guiding lanes, off-screen directional arrows, and overlaid pulsing AR beacons, which collectively assist the pilot to navigate along preplanned viewpoints accurately. The inspection quality visualization module renders the evaluated inspection metrics onto the 3D model, displaying color-coded surface maps that represent view redundancy and theoretical triangulation uncertainty. These visualizations provide immediate feedback on view redundancy and triangulation uncertainty, enabling the pilot to identify insufficiently captured regions and make informed decisions during data acquisition.

To ensure real-time processing performance and prevent UI latency, this layer utilizes Kotlin coroutines [57], a concurrency framework that enables lightweight asynchronous task execution without blocking the main thread. This design allows computationally intensive operations to be efficiently offloaded to background threads while maintaining responsive UI updates. The system follows an event-driven architecture, where processing is triggered by callbacks associated with the drone’s telemetry updates. High-frequency and latency-sensitive tasks, such as rendering AR-based viewpoint cues and updating the virtual camera view of the 3D reference model, are executed on the main UI thread to ensure smooth visualization. In contrast, computationally intensive operations, including mesh-based coverage estimation and 3D-to-2D spatial projection, are dispatched to background threads (e.g., Dispatchers.Default and Dispatchers.IO) to avoid blocking the UI pipeline. This separation of responsibilities between the UI and background threads ensures efficient resource utilization and enables stable real-time performance on resource-constrained mobile platforms.

### 2.2. Coordinate Transform and Viewpoint Projection

**Figure 3:**
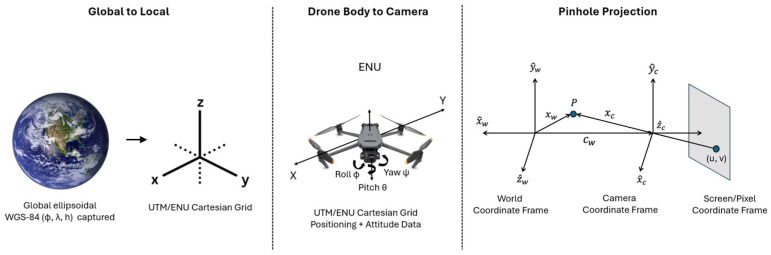
Coordinate transform from global ellipsoidal WGS-84 to local screen coordinates.

The central capability of the assistive navigation system is to guide the pilot toward preplanned inspection viewpoints. This requires projecting 3D real-world coordinates onto the 2D image plane of the remote controller’s video feed, as illustrated in Figure 3. First, the drone’s real-time global position, obtained from the RTK-GNSS module in WGS-84 geodetic coordinates (latitude, longitude, altitude), is converted into the Universal Transverse Mercator (UTM) coordinate system to enable metric spatial representation. Next, the UTM coordinates are aligned with the 3D reference model. Since the model is defined in its own local coordinate system, the system leverages embedded georeferencing information to establish a common local East-North-Up (ENU) Cartesian frame. By anchoring both the drone position and the target viewpoints to this shared reference, the relative translation vector between the drone and the target viewpoint is computed in the physical environment. Subsequently, this relative position is transformed into the camera coordinate frame to reflect the pilot’s perspective. Using real-time attitude measurements (pitch, roll, and yaw) from the gimbal and magnetometer, the ENU coordinates are aligned with the camera frame based on the sensor configuration. Finally, a standard pinhole camera model is applied to project the 3D camera-frame coordinates onto the 2D image plane using the camera’s intrinsic parameters (e.g., focal length and principal point), yielding pixel coordinates for on-screen guidance. The detailed formulation of the coordinate transformation pipeline is provided in Appendix A.

### 2.3. Augmented Reality Markers

**Figure 4:**
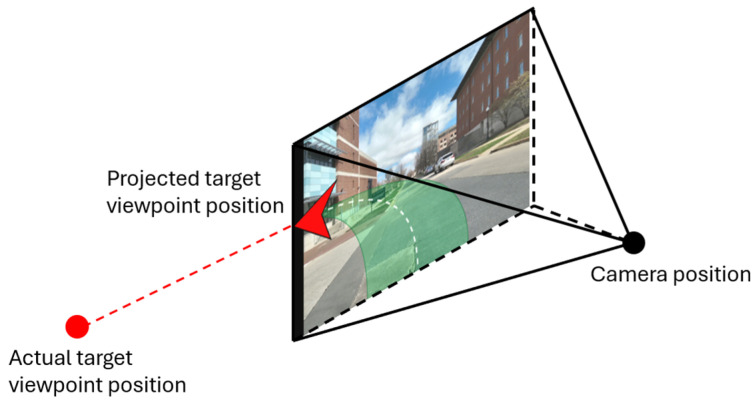
Demonstration of the target viewpoint outside the FOV projected to the image boundary.

**Figure 5:**
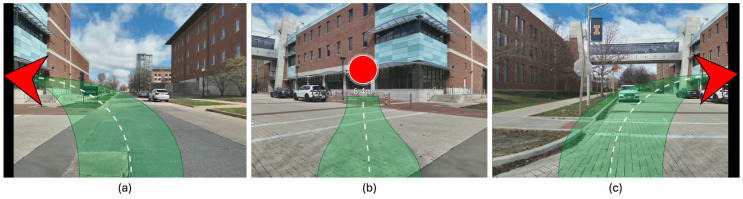
Screenshot of the target viewpoint being (**a**) off the screen to the left, (**b**) on screen, and (**c**) off the screen to the right.

Once the target viewpoint is transformed into the camera coordinate system, a *dynamic clamping* strategy is applied to generate different visual cues depending on whether the target lies within or outside the current Field of View (FOV) of the camera. As shown in Figure 4, when a target viewpoint is outside the current FOV or behind the camera (i.e., $z_{c}\leq 0$), the direction vector from the camera to the target is first projected onto the image plane. The intersection between this projected direction and the FOV boundary is then computed to determine the placement of visual guidance cues. A directional arrow and guiding lane are rendered at this boundary location, prompting the pilot to reorient the drone toward the target. Visual cueing for target viewpoints at different positions (i.e., outside and inside the FOV) is illustrated in Figure 5.

**Figure 6:**
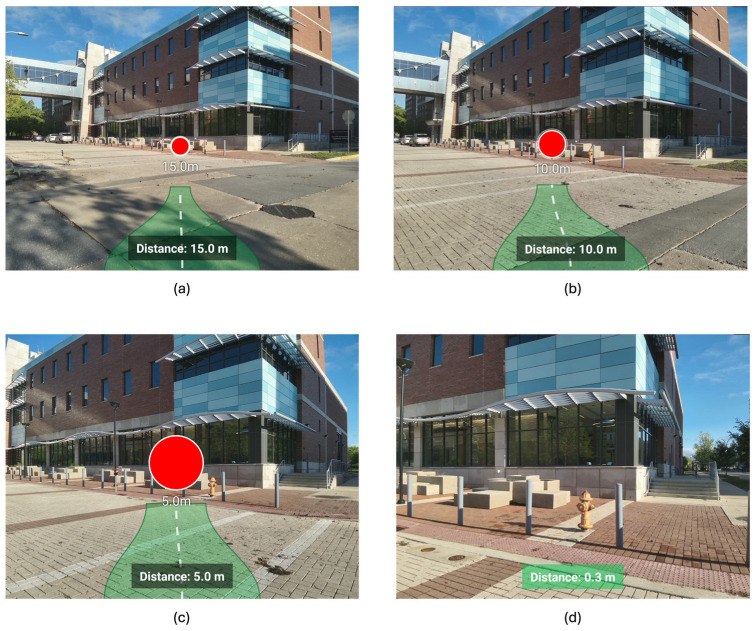
Screenshot showcasing dynamic scaling of the target viewpoint at (**a**) 15 m, (**b**) 10 m, (**c**) 5 m, and (**d**) 0.3 m, where the navigation cue disappears and the distance indicator turns green as the drone is within the distance threshold to the current viewpoint.

When the target enters the visible region, the UI transitions to display a pulsing red viewpoint beacon directly on the screen to guide the pilot towards the desired position. Besides directional guidance, the size of the beacon is dynamically scaled based on the distance between the drone and the target viewpoint, as illustrated in Figure 6, providing intuitive depth and proximity cues to assist precise positioning. When the distance falls below a predefined threshold, the red beacon disappears and the distance indicator turns green, indicating that the required positioning criteria have been met and that the system is ready for image capture.

**Figure 7:**
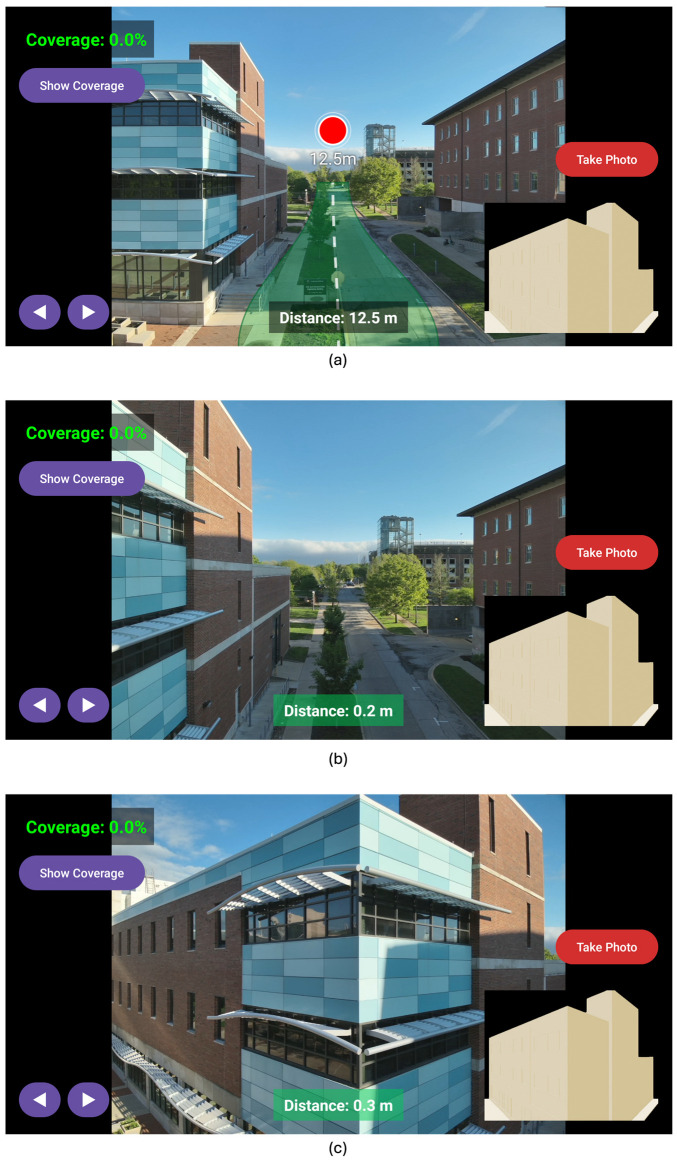
Screenshots illustrating the process of drone alignment. In (**a**), the AR viewpoint indicates the location of the current viewpoint, while (**b**) shows the navigation cues disappearing once the pilot reaches the distance threshold, and (**c**) illustrates the correct alignment of the drone based on the 3D model on the bottom right.

To further assist the pilot in adjusting the camera attitude to capture the desired image, the existing 3D model is rendered from the target viewpoint and displayed in the UI. This provides a visual reference that enables the pilot to align the drone’s orientation accordingly, as shown in Figure 7.

### 2.4. 3D Model and Coverage Estimation

**Figure 8:**
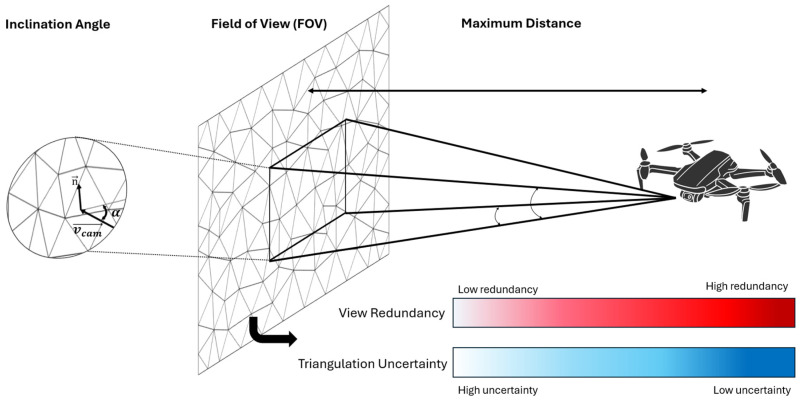
Illustration of coverage estimation with FOV, observation, and viewing distance limitations. Computation results in the view redundancy and triangulation uncertainty mesh.

To support real-time monitoring of inspection progress and data quality, the inspection quality visualization module computes mesh-coupled quality metrics, including view redundancy and theoretical triangulation uncertainty. View redundancy is a fundamental requirement for inspection tasks, as it ensures complete coverage, provides redundancy against potential image degradation (e.g., blur), and enables reliable triangulation for 3D reconstruction. In the proposed platform, the view redundancy is calculated based on multiple visibility constraints, including FOV, observation angle, viewing distance, and view occlusion, as shown in Figure 8. The detailed formulation and implementation of the view redundancy metric are provided in Appendix B.1.

These metrics are mapped onto the 3D reference model and visualized using color-coded surfaces to indicate their spatial distribution and magnitude. The visualization can be toggled between an augmented overlay on the live video stream using ARCore Sceneform [58] or through an interactive 3D model interface. This module enables the pilot to readily identify regions with insufficient data coverage or suboptimal observation geometry, thereby facilitating timely human-in-the-loop adjustments, such as capturing additional images to improve reconstruction completeness and reliability. This capability helps reduce the risk of incomplete data acquisition and minimizes the need for costly and time-consuming return visits to the inspection site.

**Figure 9:**
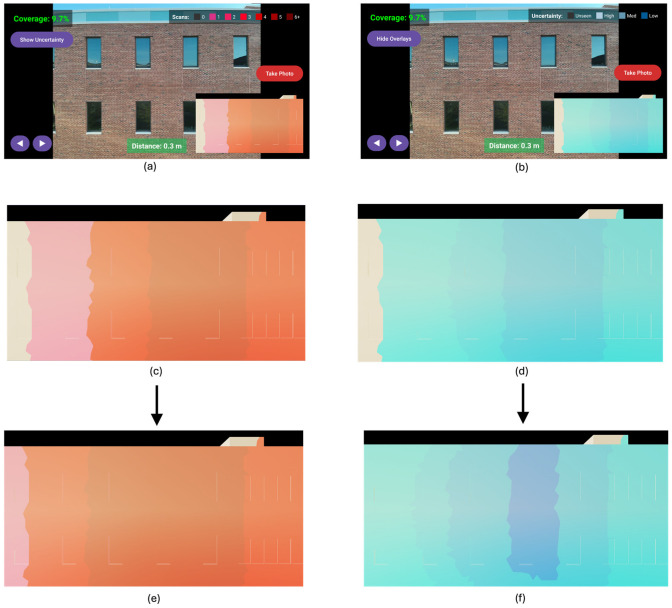
Heatmap overlaid on first-person view shown in (**a**,**b**), with (**c**,**d**) showing the redundancy and triangulation uncertainty mesh, respectively, at this step, and (**e**,**f**) showing the updated redundancy and triangulation uncertainty mesh after taking a photo. Darker colors indicate higher redundancy or lower uncertainty.

Within the 3D model interface, both the discrete view redundancy and the triangulation uncertainty can be visualized, as illustrated in Figure 9a,b. These metrics are further mapped onto an AR mesh, where they are visualized using a color-coded model to indicate the progress and quality of data acquisition. For example, prior to image capture at a given viewpoint, the corresponding view redundancy and triangulation uncertainty distributions are shown in Figure 9c and Figure 9d, respectively. After capturing an image at the current position, these metrics are updated accordingly, as shown in Figure 9e,f, reflecting the influence of the newly acquired observation. This visualization enables the pilot to identify regions with insufficient overlap or suboptimal geometric configurations and capture supplementary images to improve data completeness and reconstruction quality.

### 2.5. Human–Computer Interactions

The integration of the above subsystems results in a streamlined, human-in-the-loop workflow that balances automated guidance with manual pilot control. The full UI can be seen in Figure 2, where the operational cycle for a single viewpoint proceeds as described below.

1. **Navigation:** The pilot observes the UI guiding lane and distance metrics, then manually flies the drone into the area of the active viewpoint.
2. **Drone Alignment:** Utilizing visual cues from the 3D model within the application, the pilot fine-tunes the drone’s position and the gimbal’s orientation to match the intended reference view.
3. **Image Capture:** The pilot triggers the camera shutter using either the remote controller hardware button or the UI.
4. **Evaluation and Progression:** Upon image capture, the system updates the structural coverage mesh in real-time, enabling the pilot to verify that the captured data satisfies coverage and quality requirements before proceeding to the next viewpoint.

An important note is that the coverage estimation mesh is not updated continuously based on the real-time flight trajectory. Instead, the computational update is strictly triggered by hardware and software callbacks tied to the camera shutter. When the pilot takes a photo, the system captures the RTK GPS and Gimbal Attitude quaternion at that exact time. This design choice ensures that the coverage heatmap accurately represents the actual image data captured and saved to the device payload, preventing false-positive coverage assertions that would occur from simply panning the camera across the structure without capturing media.

## 3. Experiment Settings

This section describes the implementation details and experimental setup used to evaluate the proposed assistive navigation platform. A building façade inspection task was conducted by three pilots to compare conventional manual operation with the proposed assistive navigation approach under consistent conditions. This design ensures a fair and controlled comparison while reflecting realistic inspection scenarios.

### 3.1. Implementation Platform

The proposed assistive navigation platform is implemented following an edge-computing paradigm, with all processing executed locally on the drone’s remote controller. This design eliminates the need for continuous communication with external back-end servers during flight operations, thereby improving system robustness and reducing latency. The system is developed as an Android application using Android Studio (v2024.1.1) and the Kotlin programming language, and it integrates with the DJI Mobile SDK (MSDK v5) for real-time data access and flight control. The hardware platform consists of a DJI Mavic 3T equipped with an RTK module to provide centimeter-level positioning accuracy, paired with a DJI RC Plus 2 remote controller. The implementation’s tightly integrated hardware-software architecture enables real-time processing, reliable data acquisition, and seamless deployment in field inspection scenarios.

### 3.2. Proof-of-Concept Field Demonstration

**Figure 10:**
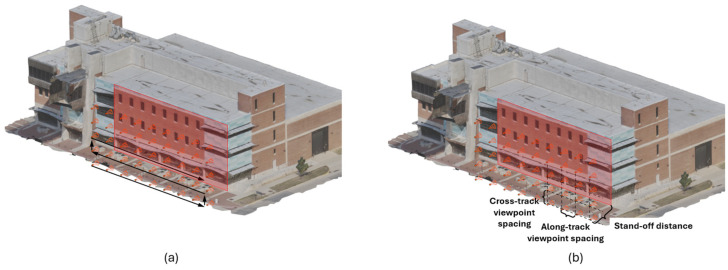
Overview of the study site, selected target inspection area, and inspection mission. (**a**) illustrates the inspection trajectory, and (**b**) presents the definition of viewpoint spacings.

To investigate the performance of the developed platform in assisting human-in-the-loop inspection, on-site reconstruction missions were conducted on the façade of the Civil and Environmental Engineering Building at the University of Illinois Urbana-Champaign (Urbana, IL, USA). Considering safety constraints related to nearby pedestrian and vehicular traffic, as well as the requirement for reliable GNSS signals, a specific region of the façade was selected as the target inspection area, as highlighted in Figure 10. Three licensed UAS pilots with Part 107 Remote Pilot Certificates were asked to perform two rounds of image collection for reconstructing the target area, one using conventional manual navigation and the other using the proposed assistive navigation platform. The manual navigation trials were conducted prior to the assistive navigation trials, and each pilot completed the experiments independently to minimize potential bias from prior exposure to the optimized mission plan.

For the manual navigation trial, the pilots were informed that the objective was to collect images suitable for SfM reconstruction of the target area. The pilots were instructed to maintain an approximate stand-off distance of 10 m from the façade, orient the camera perpendicular to the surface, and achieve approximately 70% along-track and cross-track overlap between images. No additional guidance or feedback was provided during this process. It should be noted that the 70% overlap is widely viewed as a rule of thumb for effective 3D reconstruction and has been suggested by various studies [59,60], ensuring a view redundancy of at least 9 for all surface points.

For the assistive navigation trial, a mission plan was first generated to maintain a consistent 10 m distance from the target surface while ensuring 70% along-track and cross-track overlap. The planned trajectory follows a sweep (lawnmower) pattern, as shown in Figure 10, which is commonly used in UAS-based surface inspection. The generated mission has an along-track viewpoint spacing of 4.2 m, and a cross-track viewpoint spacing of 3.1 m. During execution, the pilots were instructed to follow the real-time visual cues displayed on the user interface and capture images when approaching each target viewpoint. Image capture was triggered when the distance to the target viewpoint was below a threshold of 1 m, as indicated by a color change in the distance indicator within the interface.

### 3.3. 3D Reconstruction

To evaluate reconstruction performance under different navigation strategies, the collected images were processed in Metashape [54] to perform SfM and generate 3D models. The photo alignment accuracy was set to high, with a key point limit of 50,000 and a tie point limit of 5000, following commonly adopted default settings for reliable 3D reconstruction [61,62]. The resulting sparse point clouds were analyzed in terms of feature point density and spatial distribution to assess differences in geometric constraint quality between the navigation methods.

## 4. Results and Discussion

### 4.1. Data Collection Efficiency

The comparison of data collection efficiency is summarized in Table 1. The time required by different pilots varied considerably, largely depending on individual pilot proficiency and operational habits. All three pilots spent more time using the assistive navigation method than with manual navigation. This difference is more pronounced for Pilot 1, while relatively small for Pilots 2 and 3. This outcome is primarily attributed to the pilots’ greater familiarity and established flight habits with manual operation. In addition, the pilots noted that the momentum of the UAS made it challenging to stop precisely at designated viewpoints, requiring additional adjustments that increased the overall task duration. It is also observed that during manual navigation, pilots tend to capture more images than necessary to ensure sufficient overlap, reflecting a conservative strategy to compensate for triangulation uncertainty in viewpoint positioning. This tendency was especially noticeable for Pilot 2, while less pronounced for Pilot 1. These observations indicate the need for further improvements in user experience, control interface design, and pilot training to enhance the operational efficiency of the proposed platform.

### 4.2. Viewpoint Distribution

**Figure 11:**
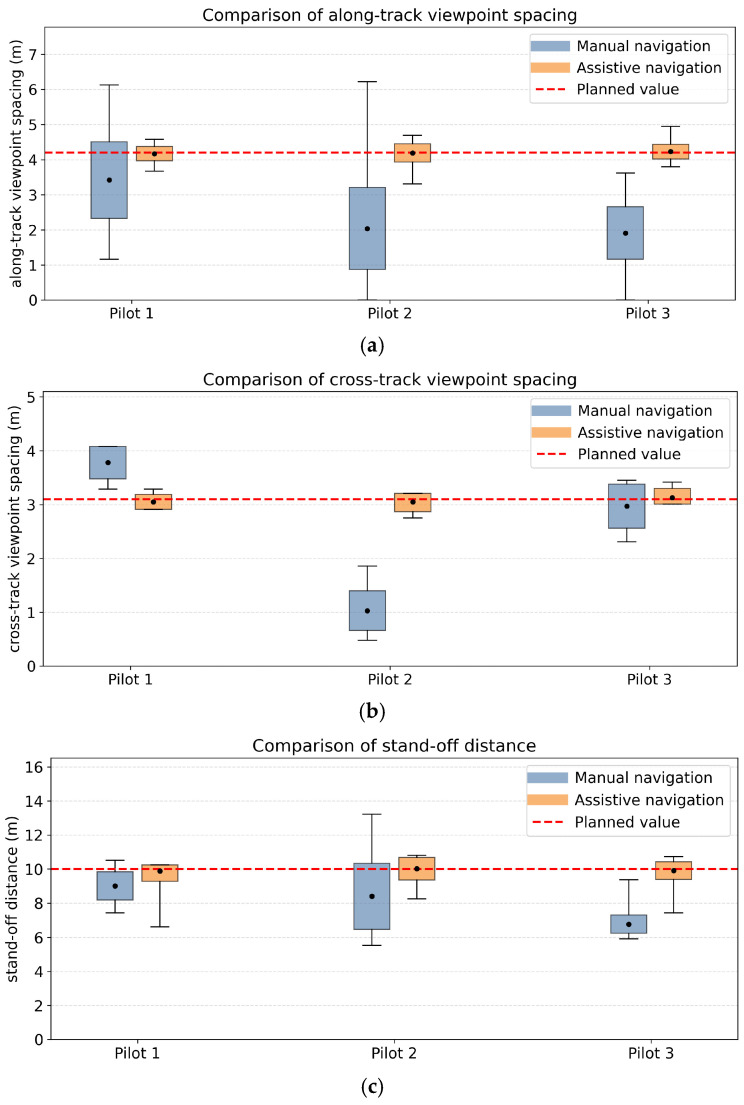
Comparative analysis of viewpoint distribution for manual and assistive navigation, showing (**a**) along-track spacing, (**b**) cross-track spacing, and (**c**) stand-off distance.

To evaluate the consistency of viewpoint distribution, along-track spacing, cross-track spacing, and stand-off distance were computed for both manual and assistive navigation, as summarized in Figure 11.

It can be observed that, for all three pilots, the assistive navigation platform achieves average along-track spacings that closely match the planned value of 4.2 m. In contrast, manual navigation results in smaller average spacings, which is consistent with the larger number of images captured during manual operation. However, manual navigation exhibits substantially higher variability, with larger maximum along-track spacings observed for Pilots 1 and 2. These variations indicate localized regions of insufficient overlap, which may lead to degraded reconstruction performance. Overall, the assistive navigation platform demonstrates the ability to enforce more controlled and consistent viewpoint sampling.

Similar trends are observed for cross-track spacing. Assistive navigation yields average values closer to the planned spacing of 3.1 m and reduced variability compared to manual navigation, further demonstrating its effectiveness in improving viewpoint distribution. Notably, the variance in cross-track spacing is generally smaller than that of along-track spacing, suggesting that maintaining lateral spacing is comparatively easier for pilots. However, the conservative image acquisition strategy observed in manual navigation is not consistently reflected across all pilots (e.g., Pilots 1 and 3), which may increase the risk of insufficient overlap in certain regions.

In addition, the stand-off distance is better maintained near the desired value of 10 m under assistive navigation. The relatively low minimum stand-off distances observed in some cases are primarily attributed to physical obstacles that blocked planned viewpoints, necessitating deviations from the intended positions. It is also observed that the variance in stand-off distance is larger than that of both along-track and cross-track spacing, indicating that controlling motion along the camera viewing direction is more challenging, even with assistive guidance. This highlights the need for further enhancement of the platform to improve visual cues for maintaining a consistent stand-off distance.

Overall, these results demonstrate that the assistive navigation approach enforces more structured and predictable viewpoint placement and distance control. Such consistency is critical for ensuring reliable image overlap and improving the quality and stability of downstream photogrammetric reconstruction, as further demonstrated in the subsequent sections.

### 4.3. View Redundancy

**Figure 12:**
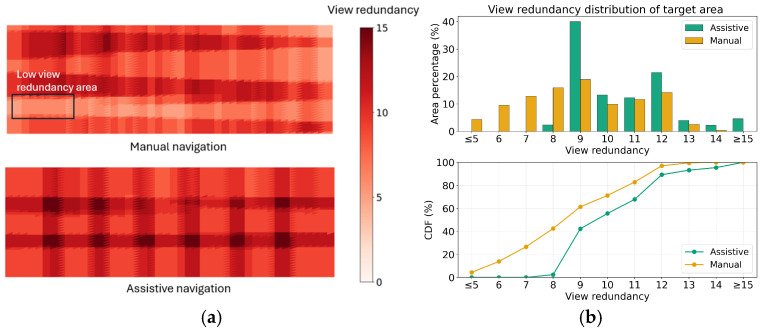
Comparison of view redundancy between manual and assistive navigation: (**a**) spatial distribution of view redundancy over the target surface; (**b**) area-weighted distribution and cumulative distribution of view redundancy values.

For 3D reconstruction tasks, view redundancy is critical for ensuring the completeness of the reconstructed model. To compare the image acquisition coverage of different methods, the images captured by Pilot 1 were used to evaluate view redundancy. A visual comparison of the spatial distribution of view redundancy over the target area is presented in Figure 12a, and the corresponding area-based distribution is shown in Figure 12b. The associated quantitative metrics are summarized in Table 2.

It can be observed from Figure 12a that assistive navigation achieves a significantly more uniform and consistent viewpoint redundancy pattern compared to manual navigation, which is consistent with the previously presented viewpoint distribution. In contrast, manual navigation resulted in a low view redundancy area, as highlighted in Figure 12a. As illustrated in Figure 12b, the cumulative distribution function (CDF) for assistive navigation lies below that of manual navigation, indicating improved performance in terms of view redundancy. Under assistive navigation, only a very small portion of faces exhibit a view redundancy of the minimum value eight, whereas in manual navigation, over 40% of faces have a view redundancy lower than eight, with a minimum value of four. This is substantially below the expected redundancy level of nine, corresponding to the targeted 70% overlap. In addition, according to Table 2, assistive navigation achieves a higher average view redundancy and lower standard deviation, indicating more consistent coverage across the inspected surface. These results suggest not only the improved uniformity provided by the assistive navigation platform but also its effectiveness in mitigating regions with insufficient view redundancy, which is a primary cause of incomplete or unstable photogrammetric reconstruction. These findings suggest that the proposed assistive navigation platform can help pilots maintain more reliable image overlap and inspection coverage during data collection, indicating the potential of improving robustness and completeness of photogrammetric inspection datasets.

### 4.4. Triangulation Uncertainty

**Figure 13:**
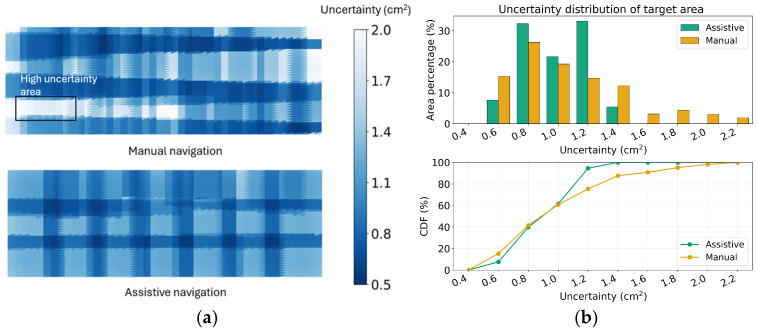
Comparison of triangulation uncertainty between manual and assistive navigation: (**a**) spatial distribution of uncertainty over the target surface; (**b**) area-weighted distribution and cumulative distribution of uncertainty values.

The theoretical triangulation uncertainty analysis further highlights the advantage of the assistive navigation approach. Similar to the view redundancy results, the triangulation uncertainty visualization for manual navigation exhibits localized regions of high uncertainty, as shown in Figure 13a, which are consistent with the previously identified areas of low view redundancy. In contrast, assistive navigation produces a more uniform distribution of triangulation uncertainty, with fewer high-uncertainty regions. A quantitative comparison is presented in Figure 13b. Both methods exhibit similar CDFs for regions with uncertainty below 1 cm^2^, while for higher uncertainty values, the CDF for manual navigation remains lower, reflecting the presence of localized high-uncertainty regions. This observation is further supported by Table 3, where assistive navigation achieves slightly lower average triangulation uncertainty, along with substantially reduced variability (standard deviation of 0.22 compared to 0.40) and a significantly lower maximum uncertainty (1.48 vs. 3.04) compared to manual navigation. Figure 13 and Table 3 indicate that by enforcing structured viewpoint placement and maintaining consistent spacing and stand-off distance, the proposed platform improves the geometric distribution of viewpoints, leading to more favorable triangulation conditions. These results indicate the potential of assistive navigation platform in enhancing the theoretical quality of the observation geometry, which is expected to contribute to more stable and reliable reconstruction outcomes.

It should be noted that the triangulation uncertainty evaluated here is a theoretical metric derived solely from the geometric configuration of viewpoint poses, rather than from the reconstructed model itself. Therefore, it reflects the expected conditioning of triangulation given the observation geometry.

### 4.5. Reconstruction Feature Point Density

**Figure 14:**
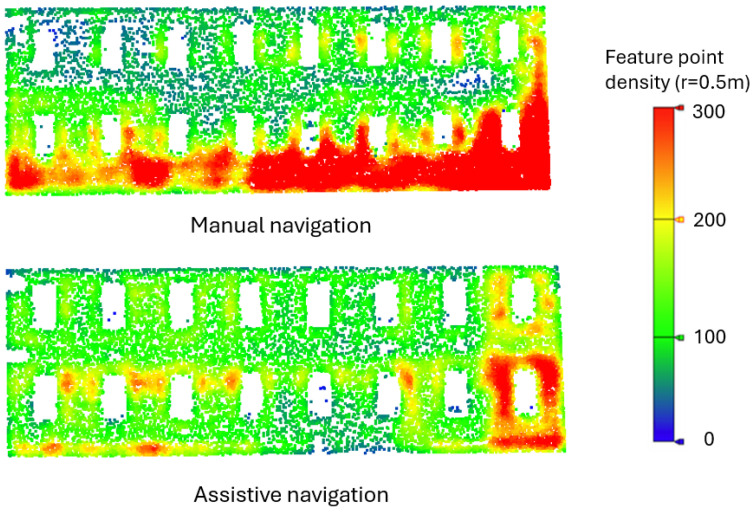
Feature point density distribution.

To evaluate the reconstruction quality achieved by different navigation methods, the collected images were processed to extract features and generate a sparse point cloud. The local point density, defined as the number of points within a 0.5 m radius, is visualized in Figure 14. A more uniformly distributed sparse point cloud indicates a more balanced spatial distribution of feature correspondences, which is essential for accurate and robust SfM reconstruction, as demonstrated in prior studies [63,64].

It can be observed that manual navigation produces an uneven distribution of feature points, with excessively high density in the lower region and significantly sparser coverage in the central and upper areas. In contrast, the assistive navigation approach achieves a much more uniform distribution across the entire surface. Although a localized high-density region is still present in the bottom-right corner, its extent is considerably smaller than that observed in the manual navigation results. More importantly, the assistive navigation method substantially reduces the area with low feature density (e.g., below a threshold of 100), indicating a lower proportion of regions with poor reconstruction quality.

### 4.6. Reconstruction Error

**Figure 15:**
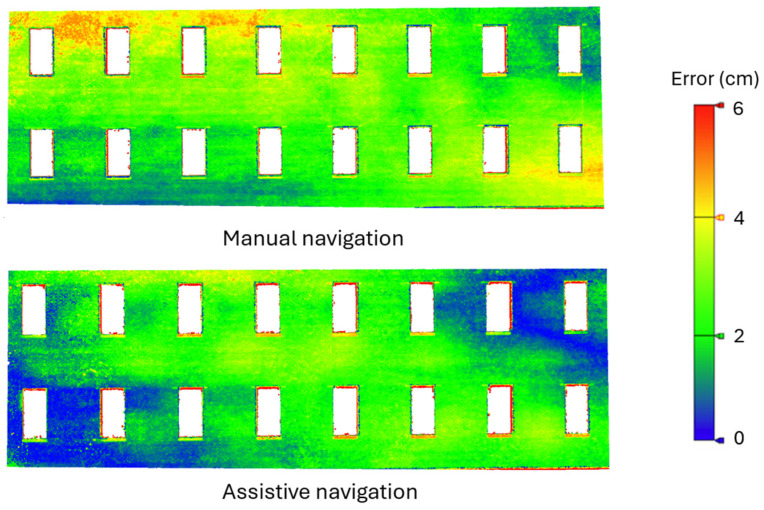
Reconstruction error distribution.

The error of the reconstructed point cloud was evaluated by calculating the distance between the reconstructed point cloud and the reference 3D model derived from the as-built drawings, which is a commonly used metric for assessing reconstruction accuracy in previous studies [15,19,65]. The distribution of reconstruction errors is shown in Figure 15.

The reconstruction generated using assistive navigation achieved an average error of 1.7 cm, compared to 2.4 cm for manual navigation, which also exhibited several localized regions with lower reconstruction accuracy. The improved performance is attributed to the more consistent viewpoint distribution and stand-off distances enabled by assistive navigation, resulting in more uniform image overlap and stable triangulation. These findings suggest that the proposed assistive navigation platform improves the quality and accuracy of photogrammetric reconstruction.

## 5. Conclusions

This study presents the system architecture for a human-in-the-loop assistive navigation platform for UAS-based infrastructure inspection that addresses the critical gap between optimized mission planning and reliable real-world execution. By integrating AR-based navigation guidance with real-time inspection quality monitoring, the proposed system enables pilots to accurately follow preplanned trajectories while maintaining awareness of data completeness and reconstruction quality.

A proof-of-concept through on-site façade inspection was conducted to assess the proposed platform. The results indicate that the platform has the potential to improve the consistency of viewpoint distribution compared to conventional manual navigation, achieving closer adherence to planned spacing and stand-off distances and resulting in more uniform spatial sampling. This improvement is associated with a more even distribution of view redundancies and fewer insufficiently covered regions.

Analysis of the reconstructed point cloud further indicates that the assistive navigation platform can reduce regions with localized low feature density and high reconstruction error. Although the proof-of-concept pilot study was limited in scale and conducted on a single inspection scenario, the results provide preliminary evidence of the potential of assistive navigation to improve inspection execution and photogrammetric data collection quality.

Overall, the proposed platform provides a practical and scalable solution for bridging the gap between automated mission planning and real-world inspection execution under current technical and regulatory constraints. By combining automated guidance with human decision-making, the system demonstrates the potential of safer, more reliable, and higher-quality infrastructure inspection.

## 6. Limitations and Future Work

While the proposed human-in-the-loop assistive navigation platform demonstrates promising results, this study primarily serves as a proof-of-concept and has several limitations. First, the field demonstration involved only three pilots evaluating a single building façade under favorable conditions. Due to the limited sample size, statistical significance analysis was not conducted, and the observed improvements should therefore be interpreted as preliminary trends rather than broadly generalizable conclusions. In addition, a relatively simple façade was intentionally selected to establish a controlled baseline, and evaluating platform robustness under challenging conditions such as wind disturbances, poor lighting, or signal degradation is outside the scope of the current study.

Future work should focus on larger-scale user studies involving more pilots, structurally complex environments, and challenging operational scenarios, including variable lighting, wind, and GNSS-denied conditions. Future studies will also incorporate human factor evaluations, such as cognitive workload assessment, to investigate whether the proposed platform can reduce pilot burden during complex inspection tasks. Further developments will aim to enhance visual guidance for more precise stand-off distance control, develop kinematic-aware predictive cueing for more accurate stopping guidance, and improve UAS maneuverability to facilitate accurate positioning at target viewpoints. In addition, integrating automated proximity alerts and collision avoidance mechanisms may further improve safety and reduce pilot workload during complex inspection tasks. The platform can also be expanded to incorporate advanced functionalities, such as defect annotation and adaptive mission refinement based on real-time inspection feedback, to improve inspection efficiency and reliability.

## Figures and Tables

**Table 1:** Comparison of data collection time and number of captured images between manual navigation and assistive navigation.

Method of Navigation	Data Collection Time (Seconds)			No. of Images		
Pilot number	1	2	3	1	2	3
Manual	220	815	405	51	206	79
Assistive	513	916	512	45	45	45

**Table 2:** Summary statistics of the distribution of view redundancy values.

Method	Average	Std	Max	Min
Manual navigation	8.99	2.14	14	4
Assistive navigation	10.58	1.80	16	8

**Table 3:** Summary statistics of the distribution of uncertainty values.

Method	Average	Std	Max	Min
Manual navigation	1.09	0.40	3.04	0.53
Assistive navigation	1.00	0.22	1.48	0.54

## Data Availability

The original data presented in the study are openly available in GitHub at https://github.com/maartoon/UAS-Inspection/ (accessed on 14 April 2026).

## Abbreviations

The following abbreviations are used in this manuscript:

UAS	Unmanned Aerial System
SfM	Structure-from-Motion
AR	Augmented Reality
UI	User Interface
UTM	Universal Transverse Mercator
ENU	East-North-Up
FOV	Field of View
GNSS	Global Navigation Satellite System
RTK	Real-Time Kinematic
CDF	Cumulative Distribution Function
BIM	Building Information Modeling

## Appendix A. Coordinate Transformation

This section details the sequential mathematical operations used to project real-world spatial coordinates onto the UAS’s 2D screen interface as described in Section 2.2.

### Appendix A.1. Geodetic to Earth-Centered, Earth-Fixed (ECEF) Transformation

The drone’s real-time Geodetic coordinates (WGS-84 format: Latitude φ, Longitude λ, Altitude h) are gathered from the RTK GNSS Module. These coordinates are then transformed into an Earth-Centered, Earth-Fixed (ECEF) grid using the standard WGS-84 ellipsoid parameters (semi-major axis a = 6,378,137.0 m, flattening f ≈ 1/298.257) using the following equations:

(A1) $$e^{2}=f\left(2-f\right)$$

(A2) $$N=\frac{a}{\sqrt{1-e^{2}\sin^{2}\left(\varphi \right)}}$$

(A3) X=(N+h)cos(φ)cos(λ)

(A4) Y=(N+h)cos(λ)sin(λ)

(A5) $$Z=\left(N\left(1-e^{2}\right)+h\right)\sin\left(\varphi \right)$$

where *e*^2^ is the square of the first eccentricity; N is the prime vertical radius of curvature; and X, Y, and Z represent the resulting ECEF Cartesian coordinates.

### Appendix A.2. Planar Projection and Local ENU Frame

Using the Proj4J library, the geodetic coordinates are mapped into the UTM coordinate system to establish a localized planar grid. To align the drone with the 3D model, the system uses the model’s georeferenced origin offsets to establish a local ENU Cartesian frame, yielding the positional vector $P_{local}=\left(x_{local},y_{local},z_{local}\right)$.

### Appendix A.3. Attitude Rotation and Coordinate Frame Transformation

To compute the viewpoint’s position relative to the drone’s perspective, the local ENU coordinates are transformed into the drone’s body frame. This is achieved by multiplying the coordinate vector $P_{local}$ by a combined rotation matrix $R_{combined}$ derived from the drone’s real-time yaw (ψ), pitch (θ), and roll (φ).

(A6) $$R_{combined}=R_{roll}\left(\psi \right)*R_{pitch}\left(\theta \right)*R_{yaw}\left(\varphi \right)$$

(A7) $$P_{drone}=R_{combined}*P_{local}$$

Because the camera sensor’s coordinate system differs from the drone’s body frame (Camera X corresponds to Drone Y, Camera Y to Drone Z, and Camera Z to Drone X), an axis-swapping transformation is applied to yield the camera-space point $P_{camera}=\left(x_{c},y_{c},z_{c}\right)$.

### Appendix A.4. Pinhole Camera Projection

Finally, a pinhole camera model projects the 3D point onto 2D pixel coordinates (u,v) utilizing the camera’s intrinsic focal lengths ($f_{x}$, $f_{y}$,) and principal point offsets $\left(c_{x},c_{y}\right)$. An illustration of the coordinate transformation from the drone’s real-time geodetic coordinates to screen coordinates on the remote controller can be seen in Figure 4.

(A8) $$u=\left(f_{x}*x_{c}/z_{c}\right)+c_{x}$$

(A9) $$v=\left(f_{y}*y_{c}/z_{c}\right)+c_{y}$$

Points where $z_{c}\leq 0$ are determined to be behind the camera and subsequently passed to the dynamic clamping algorithm to render off-screen directional arrows.

## Appendix B. Quality Metrics

### Appendix B.1. View Redundancy

View redundancy is defined as a quality metric for each face on the target model, representing the number of viewpoints from which the face is visible. It is formulated as:

(A10) $$r\left(f_{i},P\right)=\sum_{p_{j}\in P}v(f_{i},p_{j})$$

where $r\left(f_{i},P\right)$ denotes the view redundancy of face $f_{i}$ along path P, and $v(f_{i},p_{j})$ represents the Boolean visibility of face $f_{i}$ from a single viewpoint $p_{j}$ on the path.

When calculating visibility, in addition to basic constraints such as the camera FOV and potential occlusions, factors related to image quality must also be considered. For instance, excessive viewing distance or steep observation angles can degrade feature detection and matching, leading to unstable or incomplete reconstruction. Therefore, visibility is evaluated based on four criteria: FOV, observation angle, viewing distance, and view occlusion. The overall visibility is defined as:

(A11) $$v\left(f_{i},p_{j}\right)=v^{FOV}\left(f_{i},p_{j}\right)v^{oa}\left(f_{i},p_{j}\right)v^{d}\left(f_{i},p_{j}\right)v^{vo}\left(f_{i},p_{j}\right)$$

in which $v^{FOV}$, $v^{oa}$, $v^{d}$, and $v^{vo}$ denote the FOV constraint, orientation angle constraint, distance constraint, and view obstruction constraint, respectively. Each factor is Boolean, taking a value of 1 if the condition is satisfied and 0 otherwise. A face is considered visible from a viewpoint only if all four conditions are satisfied.

The calculation of each constraint is detailed below.

#### Appendix B.1.1. FOV Check

The FOV check determines whether a target point lies within the camera’s viewing frustum. The horizontal and vertical angles of the target point relative to the camera are computed as:

(A12) $$h_{angle}=arctan2\left(p\cdot r,p\cdot f\right)$$

(A13) $$v_{angle}=arctan2\left(p\cdot u,p\cdot f\right)$$

where the inputs are defined in Figure 4. The FOV visibility condition is then given by:

(A14) $$v^{FOV}=\left[h_{angle}<h_{FOV}\right]\left[v_{angle}<v_{FOV}\right]$$

in which [·] denotes the Iverson bracket, which evaluates the logical condition to 1 if true and 0 otherwise.

#### Appendix B.1.2. Observation Angle Check

The observation angle between target face $f_{i}$ and viewpoint $p_{j}$ is defined as the angle between the face normal $f_{i}^{n}$ and viewing direction $r_{ij}$, in which $r_{ij}$ indicates the vector from viewpoint to target face. Although a face may lie within the camera’s FOV, a large observation angle can degrade image quality and adversely affect feature detection and matching, leading to unreliable reconstruction. Therefore, an observation angle constraint is introduced to ensure sufficient visual quality:

(A15) $$v^{oa}\left(f_{i},p_{j}\right)=\left[\frac{-f_{i}^{n}r_{ij}}{\left\|f_{i}^{n}\right\|\left\|r_{ij}\right\|}>\cos\alpha \right]$$

where α is the maximum allowed observation angle. Note that the negative sign in the numerator reverses the segment as $f_{i}^{n}$ originates from the target face.

#### Appendix B.1.3. Distance Check

By applying distance check, the feature points that are too far from a viewpoint will not be regarded as visible. The distance check is shown in Equation (A16):

(A16) $$v^{d}\left(f_{i},p_{j}\right)=\left[\left\|r_{ij}\right\|<d_{t}\right]$$

in which $d_{t}$ is the distance threshold.

#### Appendix B.1.4. View Obstruction Check

To ensure that no obstacles exist between the viewpoint and the target face, the Möller–Trumbore intersection algorithm [66] is employed to determine whether the line segment between the viewpoint and the target intersects any other faces in the scene. The visibility term $v^{vo}\left(f_{i},p_{j}\right)$ is assigned a value of 1 if no obstruction is detected, and 0 otherwise.

Although the Möller–Trumbore algorithm performs ray–triangle intersection testing in constant time, the overall computational cost becomes prohibitive when applied exhaustively, as each visibility query requires testing against all faces in the model. As a result, the total complexity scales quadratically with respect to the number of faces, leading to substantial computational overhead. To optimize the algorithm for real-time mobile execution, a hierarchical filtering strategy is employed. Specifically, other computationally inexpensive visibility checks are first applied to prune the invisible faces. The more computationally intensive obstruction test is then performed only on this reduced set of faces, thereby significantly lowering the overall computational cost and improving execution efficiency.

### Appendix B.2. Triangulation Uncertainty

The triangulation uncertainty formulation follows the derivation in [67]. For a given face $f_{i}$, the information provided by path P is quantified using the Fisher Information Matrix H(P, $f_{i}$), which aggregates the geometric contributions of all viewpoints from which the face is visible. Specifically, the matrix H is formulated as:

(A17) $$H(P)=\sum_{p_{j}\epsilon P}\frac{\left(I-r_{ij}*r_{ij}^{T}\right)}{{\left|\left|r_{ij}\right|\right|}^{2}}v(f_{i},p_{j})$$

where I is the 3 × 3 identity matrix, $p_{j}$ denotes a viewpoint along path P, $r_{ij}$ is the vector from the viewpoint $p_{j}$ to target face $f_{i}$, and $v(f_{i},p_{j})$ is the binary visibility indicator defined in Appendix B.1. The summation is taken over all viewpoints from which the target face is visible.

The triangulation uncertainty is then quantified by the trace of the inverse of H. Incorporating the base sensor noise with variance $\sigma^{2}$, the final triangulation uncertainty metric is given by:

(A18) $$Uncertainty=\sigma^{2}*Trace\left(H^{-1}\right)$$

It should be noted that the variance $\sigma^{2}$ is only related to the camera sensor instead of the trajectory. Therefore, the calculated triangulation uncertainty is a relative metric with an assumed variance. In our work, we assume 0.001 for this value, which corresponds to 1 mm reprojection error at 1 m distance, or approximately three pixels reprojection error for 4000 px × 3000 px image with horizontal FOV of 70° and vertical FOV of 55°.
